# Integrated transcriptomic and metabolomic analyses of yellow horn (*Xanthoceras sorbifolia*) in response to cold stress

**DOI:** 10.1371/journal.pone.0236588

**Published:** 2020-07-24

**Authors:** Juan Wang, Jinping Guo, Yunxiang Zhang, Xingrong Yan

**Affiliations:** 1 College of Forestry, Shanxi Agricultural University, Taigu, Shanxi, China; 2 Shanxi Key Laboratory of Functional Oil Tree Cultivation and Research, Taigu, Shanxi, China; ICAR - National Research Center on Plant Biotechnology, INDIA

## Abstract

*Xanthoceras sorbifolia*, a medicinal and oil-rich woody plant, has great potential for biodiesel production. However, little study explores the link between gene expression level and metabolite accumulation of *X*. *sorbifolia* in response to cold stress. Herein, we performed both transcriptomic and metabolomic analyses of *X*. *sorbifolia* seedlings to investigate the regulatory mechanism of resistance to low temperature (4 °C) based on physiological profile analyses. Cold stress resulted in a significant increase in the malondialdehyde content, electrolyte leakage and activity of antioxidant enzymes. A total of 1,527 common differentially expressed genes (DEGs) were identified, of which 895 were upregulated and 632 were downregulated. Annotation of DEGs revealed that amino acid metabolism, glycolysis/gluconeogenesis, starch and sucrose metabolism, galactose metabolism, fructose and mannose metabolism, and the citrate cycle (TCA) were strongly affected by cold stress. In addition, DEGs within the plant mitogen-activated protein kinase (MAPK) signaling pathway and TF families of ERF, WRKY, NAC, MYB, and bHLH were transcriptionally activated. Through metabolomic analysis, we found 51 significantly changed metabolites, particularly with the analysis of primary metabolites, such as sugars, amino acids, and organic acids. Moreover, there is an overlap between transcript and metabolite profiles. Association analysis between key genes and altered metabolites indicated that amino acid metabolism and sugar metabolism were enhanced. A large number of specific cold-responsive genes and metabolites highlight a comprehensive regulatory mechanism, which will contribute to a deeper understanding of the highly complex regulatory program under cold stress in *X*. *sorbifolia*.

## Introduction

Abiotic stresses unfavorably affect growth and productivity in plants and cause a series of changes at the morphological, physiological, biochemical, and molecular levels [1–3]. Low temperature (LT) is one of the major abiotic stresses in plants that disturbs physiological, cellular, metabolic, and molecular functioning, resulting in severe retardation of plant growth and development, and frequently even death [4]. Cell membranes may become disorganized, osmotic stress can be altered, proteins may lose activity or be denatured, and high levels of reactive oxygen species (ROS) can damage the oxidation system [5]. Further, ROS can trigger various signaling pathways, such as mitogen-activated protein kinase (MAPK) signal transduction cascades for specific responsive gene expression. ROS are limited or scavenged by antioxidant enzymes, such as superoxide dismutase (SOD), peroxidase (POD), and catalase (CAT) [6]. During cold-stress response, plant cells tend to accumulate a series of osmoregulatory metabolites, including soluble sugars (e.g., sucrose, glucose, and galactose) and low molecular weight compounds (e.g., proline, glycine, and betaine) that enable plants to alleviate osmotic stress and maintain cell turgor, water uptake, and metabolic activity [7, 8].

Numerous studies have demonstrated that cold tolerance is a complex quantitative trait involving multiple regulatory mechanisms and metabolic pathways, and is associated with activities of antioxidant enzymes, expression of cold-responsive genes and transcription factors (TFs), and the concentrations of primary and secondary metabolites [9, 10]. Plants can respond to cold stress through a wide range of transcriptional changes to maintain cellular homeostasis and improve cold tolerance. For instance, previous studies have indicated that the CBF transcriptional network plays a central role in cold response in *Arabidopsis* [11]. Exposing *Arabidopsis* plants to LT can rapidly induce a family of three transcriptional activators (CBF1-3) that specifically bind to the CRT/DRE (C-repeat/dehydration-responsive element) *cis*-acting regulatory element of the promoter region of many cold-inducible genes, present in the promoters of COR15a and COR78/RD29a [12]. Therefore, transcriptome analysis is a powerful technology for gene-expression profile analysis at the whole-genome level that has been used to identify numerous cold-responsive genes from different species, including *Arabidopsis* [13, 14], maize [15, 16], rice [17–19], grape [20], and peach [21]. In response to LT stimuli, plants evolve an array of metabolic responses oriented toward stress avoidance, defense, or resistance depending on the particular stress tolerance. Among all metabolic responses, alterations to primary metabolism are the most evident and involve changes in levels of sugars and sugar alcohols, amino acids, and TCA cycle intermediates, thereby exhibiting general trends in response to cold stress. However, changes in secondary metabolism are more species-specific [22]. At present, metabolomic studies have also been applied to explore metabolites involved in cold-stress regulation in many plants, such as maize [23], spruce [24], orange [25], and *Arabidopsis* [26]. Meanwhile, transcriptomics integrated with metabolomics can contribute to a deeper understanding of the gene-to-metabolite pathways associated with cold stress in plants.

*Xanthoceras sorbifolia*, commonly known as yellow horn, is a member of the Sapindaceae family and originates in China [27]. It is a woody deciduous shrub or small tree with a lifespan of more than 200 years [28]. Yellow horn is considered an important bio-energy feedstock plant because of the significant concentration of oil in the seed kernel (> 60%), and it may become an energy-sustainable substitute for petroleum. Furthermore, the oil contains unsaturated fatty acids up to 90.9% of the total fatty acids, which have potential health benefits [29]. It is highly resistant to environmental stresses, such as cold, drought, and salt stresses. The chloroplast genome of *X*. *sorbifolia* has been assembled and characterized [30]. The whole genome of *X*. *sorbifolia* has been sequenced and assembled recently [31, 32]. There have been some reports on the investigations into *X*. *sorbifolia* transcriptome, including oil accumulation [33, 34], fertilized ovule development [35], as well as abiotic stresses (e.g., NaCl, ABA and low temperature) [36]. However, few studies have focused on the comprehensive information based on transcriptional and metabolic responses to cold stress in *X*. *sorbifolia*.

In this study, we integrated the transcriptomic and metabolomic analyses of *X*. *sorbifolia* seedlings to explore the changes that occur in the transcription and metabolite levels in response to cold stress, and sought to reveal potential links between the expression of cold-responsive genes and metabolites accumulation. Therefore, a gene-to-metabolism network constructed on the basis of observations of massive transcriptome and metabolome reprogramming can further elucidate a comprehensive gene regulatory mechanism that involves cold-signal perception and transduction, transcriptional regulation, and gene expression. These findings could provide us with a unique opportunity to gain novel insights into the molecular mechanisms regulating cold tolerance in *X*. *sorbifolia*.

## Materials and methods

### Plant materials and treatments

*X*. *sorbifolia* seeds were collected from the same tree (accession number: A099), which had several superior traits including high oil content, high yield and a certain resistance to cold stress. This tree was grown from the breeding base owned by Shanxi Agricultural University (37°25′N, 112°34′E) in China. Seeds were germinated with the sand-hiding method at 25 °C because the seed coat is thick and hard. After 20 days, seedlings were grown in a greenhouse (light 25 °C for 12 h/dark at 20 °C for 12 h; relative humidity of 60% –70%) and regularly watered under natural conditions for a month. From mid-April to mid-May, the changes in local temperature are often unstable, and sometimes low temperature (about 4 °C) lasts for one to several days, which can affect the normal growth of *X*. *sorbifolia* seedling. We thus hypothesized that the changes should also occur in the transcription and metabolite levels of *X*. *sorbifolia* after cold stress at 4°C. Healthy and uniform seedlings (height, ~30 cm) were treated at 4 °C at different time points. The leaves of these seedling plants were harvested at 0 (control), 4, 12, and 24 h for transcriptome sequencing, and the same samples of 0 (control) and 24 h were prepared for metabolite profiling with three biological replicates per treatment. We selected 24 h as the extreme time point according to the phenotypic changes of *X*. *sorbifolia* seedlings after cold treatment compared with control (0h). All samples were frozen in liquid nitrogen immediately and stored at −80 °C until further use.

### RNA extraction, cDNA synthesis, and sequencing

Total RNA from each sample was extracted using the TaKaRa MiniBEST Plant RNA Extraction Kit (TaKaRa, Dalian, China) following the manufacturer’s instructions. The concentration and purity of the extracted RNA was verified by agarose gel electrophoresis (1%) and a NanoDrop 2000C spectrophotometer (Thermo Fisher Scientific, Wilmington, USA). The integrity of RNA was assessed with an Agilent Bioanalyzer 2100 (Agilent Technologies, Santa Clara, USA). Sequencing libraries were created using the NEB Next^®^ Ultra^™^ RNA Library Prep Kit for Illumina^®^ following the manufacturer’s instructions. Index codes were added to each sample. The prepared libraries were sequenced on the Illumina HiSeq 2500 platform (Illumina Inc., San Diego, USA) by Biomarker Technology Corporation (www.biomarker.com.cn). The raw RNA-Seq data were submitted to www.ncbi.nlm.nih.gov/bioproject/PRJNA608707.

### Transcriptome assembly and function annotation

The transcriptome was assembled from all 12 samples, including 3 control samples and 9 cold-stressed samples. The clean reads were obtained after removing low-quality reads, adapter sequences, and reads containing poly-N from the raw data. The high-quality reads were assembled *de novo* into transcripts using Trinity software with the paired-end method. All assembled unigenes were annotated in Kyoto Encyclopedia of Genes and Genomes (KEGG) [37], Gene Ontology (GO) [38], Clusters of Orthologous Groups (COG) [39], eggnog [40], Swiss-prot [41], NR [42], and euKaryotic Orthologous Groups (KOG) [43] using BLAST [44] with E-value ≤ 1.0 × 10^−5^. Transcription levels for each gene were quantified according to the FPKM (fragments per kilobase of exon per million fragments mapped). The FPKM was calculated by normalizing for the length of the gene and total read number mapped to it [45, 46]. False discovery rate-adjusted *P* value (FDR) < 0.05 and an absolute value of log2 FC ≥ 1 were used as the empirical parameters to identify the differentially expressed genes (DEGs). The KEEG pathway annotation was performed on the KAAS (KEEG Automatic Annotation Serve) website (http://www.genome.jp/tools/kaas/). GO annotation was performed using BinGo (https://www.psb.ugent.be/cbd/papers/BiNGO/Home.html) by assigning the annotation to *Arabidopsis* (*P* value < 0.05), and networks of enriched GO terms were constructed with Cytoscape 3.7.2. TFs were predicted using the PlantTFDB database (http://planttfdb.cbi.pku.edu.cn/).

### Metabolic profiling analysis

Frozen samples were sent to Biomarker Technologies Co., Ltd. (Beijing, China) for extract analysis, metabolite identification, and quantification following their standard procedures and previously described by Chen et al. [47]. Briefly, the samples were dried and ground to powder, and powdered samples were extracted and purified by filtration and centrifugation. The purified solution was injected into the GC-TOF-MS system (Pegasus HT, LECO, San Jose, USA) for analysis. The metabolite separations were performed using an Agilent DB-5MS capillary column (30 m×250 μm×0.25 μm, J&W Scientific, Folsom, USA). Mass spectral data were analyzed using ChromaTOF V4.3x (LECO San Jose, USA), including peak extraction, baseline correction, deconvolution, peak integration, and peak alignment. Metabolites were quantified by comparison to the internal standard values and identified according to the LECO-Fiehn Rtx5 database. The data matrices of identified metabolites from 6 samples were uploaded to the Metaboanalyst (http://www.metaboanalyst.ca/) for statistical and pathway analyses. Differential metabolic features were defined according to fold-change > 1.5, variable importance in projection VIP > 1 of the partial least-squares discriminant analysis (PLS-DA) [48], and statistically significant (*P* value < 0.05) based on Student’s t-test.

### Physiological measurements

The leaves were sampled and physiological indices were measured after 0 (control), 4, 12, 24, and 48 h of the 4 °C treatment. All assays were conducted with three biological repeats. For measurement of electrolyte leakage (EL), the leaf samples were put into 30 ml distilled water and shaken at room temperature for 3 h. The initial conductivity (E1) was measured using a digital conductivity meter. Then, the samples were boiled for 30 min and cooled to room temperature, the final electrolyte conductivity (E2) was measured. Electrolyte leakage (%) = (E1/E2) × 100. The malondialdehyde (MDA) accumulation was measured by the thiobarbituric acid (TBA) colorimetric method using an MDA assay kit (Solarbio, Beijing, China). The content of MDA was expressed as μmol MDA per gram FW.

Activities of SOD, POD, and CAT were determined according to instructions from commercially available kits (Solarbio, Beijing, China). Fresh leaves (0.5 g) were ground to a fine powder in liquid nitrogen, and enzymes were extracted using extraction buffer. Subsequently, the extraction was centrifuged at 8,000 × g for 10 min at 4 °C, and the supernatant was used for further experiments. The final reacted solutions were determined using spectrophotometry at 560 nm (for SOD activity), 470 nm (for POD activity), and 240 nm (for CAT activity). All measurements were performed according to the instructions of the antioxidant enzyme assay kit. The enzyme activities were expressed as units of enzyme activity per gram FW.

Statistical analysis was conducted with analysis of variance (ANOVA) and Student’s t-test using the Statistical Package for the Social Sciences (SPSS) software (IBM SPSS Statistics, version 21.0, Armonk, USA).

### Quantitative real-time PCR (qRT-PCR) analysis

qRT-PCR experiments were carried out with the same RNA samples for the transcriptome analysis on the ABI 7500 (Applied Biosystems, Carlsbad, USA). *X*. *sorbifolia Actin* (c24784.graph_c0) was used as an internal control. Specific primer pairs of selected genes were designed by Primer 5.0 with the primer sequences listed in S1 Table. Each reaction was performed in a 20-μL mixture using the TB Green^®^ Premix Ex Taq^™^ II (TaKaRa, Dalian, China) according to the manufacturer’s instructions. The relative expression levels of the genes were analyzed with ABI 7500 Software V2.3, and quantified using the 2^−ΔΔCt^ method, which represents the cycle threshold (CT) of the target gene relative to the reference *Actin* gene. Three independent biological replicates were used for qRT-PCR analysis.

## Results

### Malondialdehyde content, electrolyte leakage and antioxidant enzyme activity

**Fig 1:**
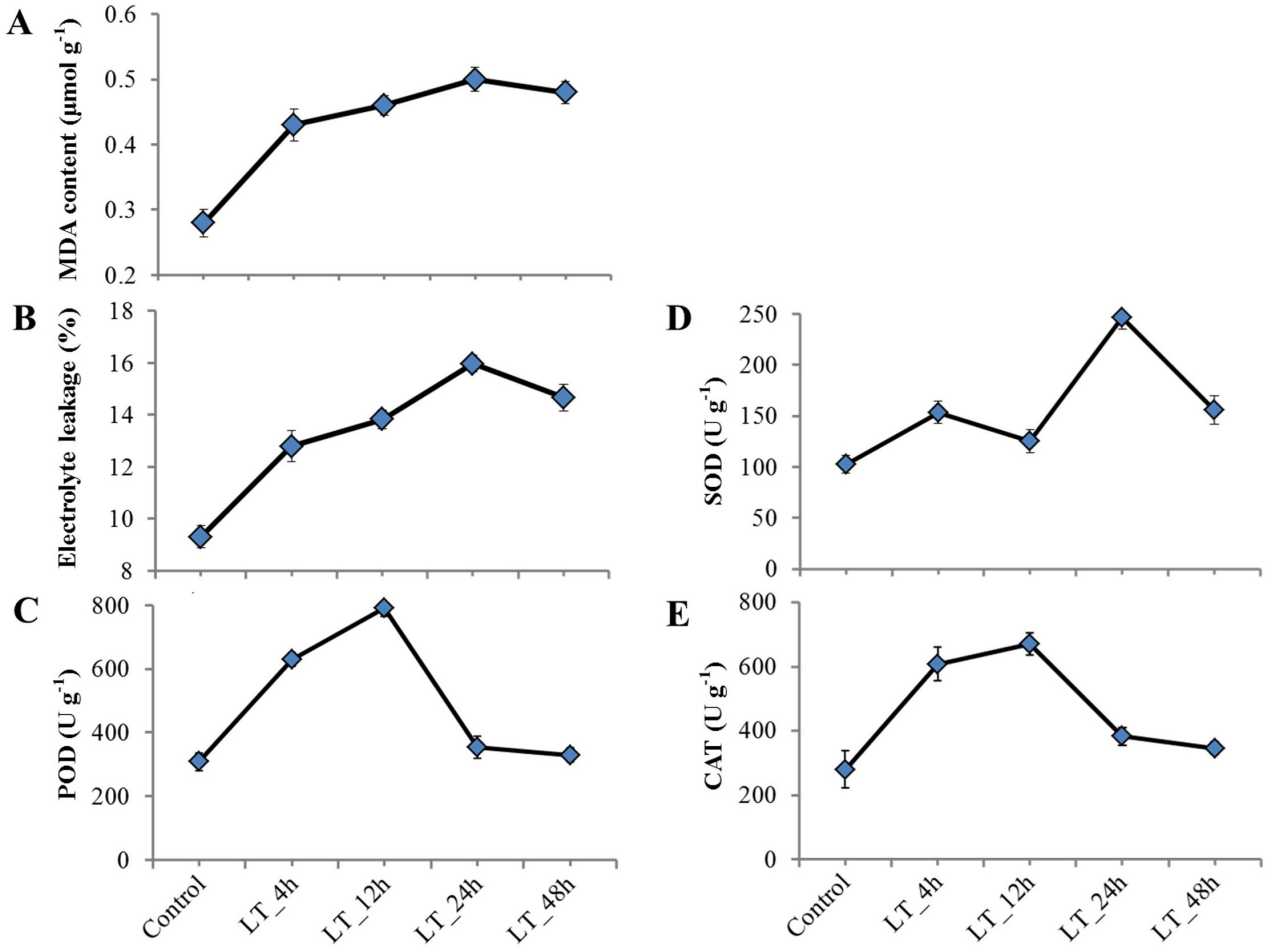
Changes in malondialdehyde content, electrolyte leakage and antioxidant enzyme activity in leaf tissues of *X*. *sorbifolia* under cold stress. (A) Malondialdehyde (MDA) content, (B) electrolyte leakage (EL), (C) Peroxidase (POD), (D) Superoxide Dismutase (SOD), and (E) Catalase (CAT). Error bars indicate means ± SD (n = 3).

MDA and EL levels are commonly used to assess the degree of membrane injury [49]. After low temperature stress, MDA content and EL value were significantly increased relative to the control, and they showed the same trend with the extension of the stress time (Fig 1A and 1B). MDA content and EL value increased rapidly within the first 4 h, then further increased slightly and reached peak level at 24 h, but subsequently decreased slowly until 48 h.

Compared to the control, lowering the temperature caused a general increase in the activities of all three enzymes in leaf tissue (Fig 1C–1E). The enzyme activities appeared a follow “rise to drop” tendency as time progressed. POD and CAT activity almost linearly increased during the first 12 h, and thereafter decreased sharply to the lowest level at 48 h, but it still higher than the control value. SOD activity initially increased slowly until 12 h, then rose rapidly and reached peak level at 24 h, but diminished sharply thereafter.

### Transcriptome

The RNA-Seq analysis yielded approximately 92.53 Gb clean reads from 12 transcriptome libraries, on average 6.35 Gb for each library. Over 85% of the sequences in each library were mapped, indicating that the assembled transcriptome was available for differential expression analysis. The value of ≥Q30 was used to assess the quality of sequencing, and 93.65–94.38% of bases scoring Q30 for each library suggested that the RNA-Seq datasets are of high quality (Table 1). A total of 63,197 unigenes were obtained with an average length of 1,077 bp and N50 of 2,472 bp after assembly and data analysis, including 17,643 unigenes with lengths over 1 kb (S2 Table). The correlation heatmap analysis (S1 Fig) revealed that the 3 biological replicates showed high reproducibility for all treatments, and the gene expression data were closely related at each time point. In total 13,675, 13,544, 13,229, and 12,945 genes were expressed in the “Control”, “LT_4h”, “LT_12h”, and “LT_24h”, respectively, and a summary of the DEGs between samples at different time points is found in S2A Fig. The Venn diagram (S2B Fig) exhibits a total of 11,724 genes being expressed in all 5 groups, while 1,213 genes were specifically expressed in only 1 group (“Control”, 549 genes; “LT_4h”, 230 genes; “LT_12h”, 142 genes; “LT_24h”, 292 genes).

**Table 1:** Overview of the transcriptome sequencing data.

Sample	Raw reads	Clean reads	Mapped reads	GC (%)	≥Q30 (%)
Control_1	7,171,112,530	24,041,522	20,842,522 (86.69%)	46.37	94.38
Control_2	8,368,100,334	28,077,693	24,279,069 (86.47%)	46.18	94.33
Control_3	9,418,103,704	31,593,500	27,360,921 (86.6%)	46.14	94.32
LT_4h_1	7,646,608,490	25,633,555	22,068,145 (86.09%)	47.17	94.20
LT_4h_2	8,899,831,612	29,829,113	25,835,520 (86.61%)	46.32	93.98
LT_4h_3	7,742,704,904	25,948,842	22,460,971 (86.56%)	46.25	93.94
LT_12h_1	6,896,939,568	23,136,097	19,954,286 (86.25%)	46.62	93.91
LT_12h_2	6,416,910,880	21,481,446	18,392,693 (85.62%)	46.06	93.90
LT_12h_3	8,452,995,722	28,341,540	24,367,088 (85.98%)	46.37	93.87
LT_24h_1	6,351,545,286	21,288,261	18,243,642 (85.7%)	46.03	93.82
LT_24h_2	7,906,165,170	26,511,245	22,833,381 (86.13%)	46.36	93.72
LT_24h_3	7,258,175,670	24,333,119	20,914,402 (85.95%)	46.33	93.65

### Functional annotation

**Fig 2:**
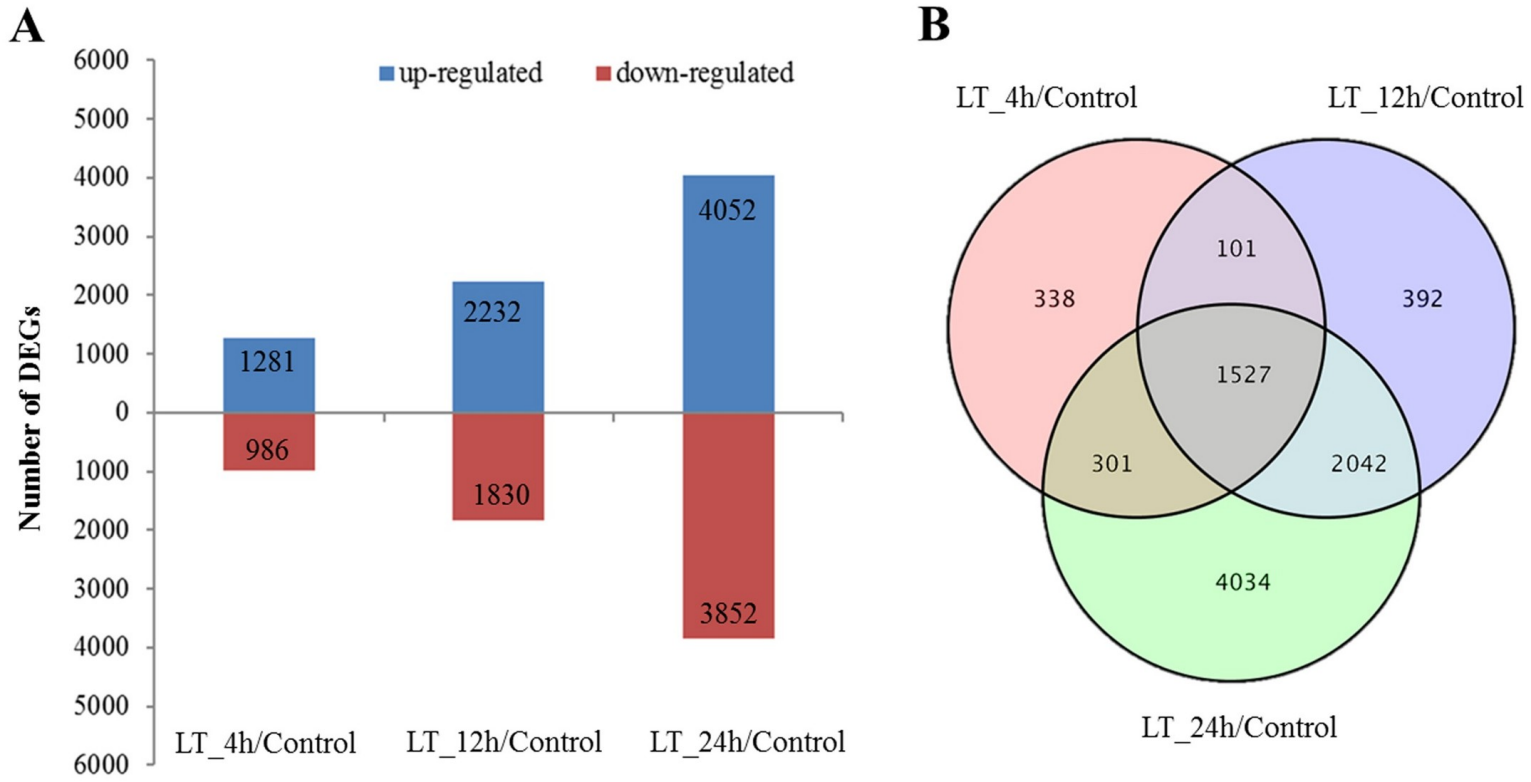
DEGs in the three pairwise comparisons of the control and stress treatments. (A) Bar chart showing the number of up- and downregulated genes in different comparisons. (B) Venn diagram showing the common and specific DEGs in different comparisons.

Each stress sample was compared with the control to identify DEGs. We identified 2,267 (1,281 up- and 986 downregulated) DEGs in the LT_4h sample, 4,062 (2,232 up- and 1,830 downregulated) DEGs in the LT_12h sample, and 7,904 (4,052 up- and 3,852 downregulated) DEGs in the LT_24h sample (Fig 2A), revealing that the number of upregulated DEGs was much higher than the number of downregulated DEGs. A total of 1,527 common DEGs, including 895 up- and 632 downregulated DEGs were identified and used for functional annotation (Fig 2B).

### TF prediction

**Fig 3:**
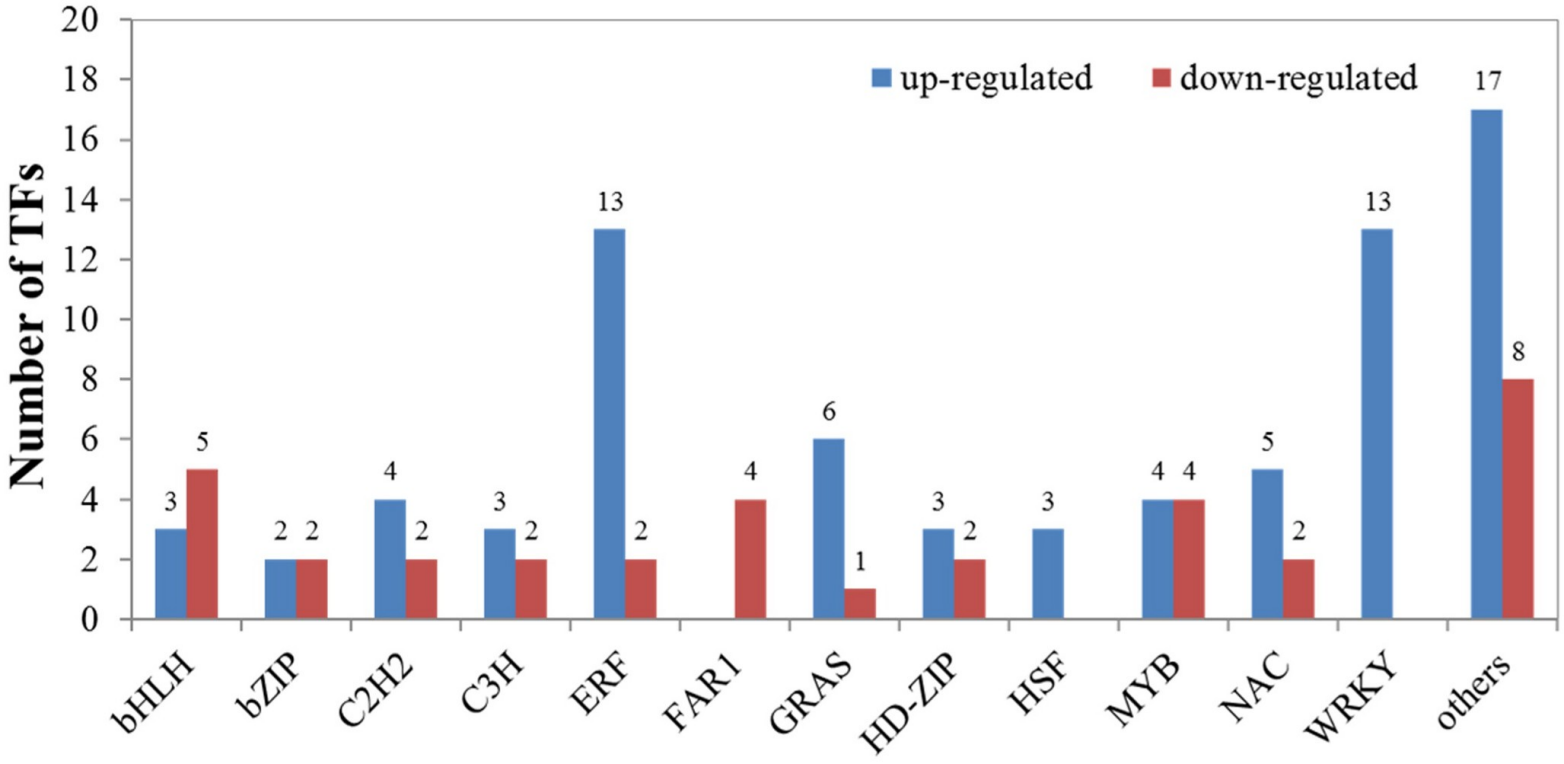
Distribution of the DEGs into different transcription factor families during cold stress. Blue and red colors indicate up- and downregulated transcripts, respectively.

Based on all 81 families of TFs predicted from the *Arabidopsis* TF database [50], we detected 32 families with at least 1 gene matched to the DEG dataset. In total, 110 DEGs as TFs were identified in seedling leaves of *X*. *sorbifolia* in response to cold stress, including 76 up- and 34 downregulated TFs (Fig 3, S3 Table). Most of the TF families had more upregulated TFs than those that downregulated during cold stress compared with the control. In contrast, certain TFs belonging to the FAR and bHLH families showed more downregulation. In addition, WRKY, bHLH, ERF, MYB, and NAC families featured more active members, implying their important roles in the regulation of structural genes involved in cold response.

### KEGG pathway classification

**Fig 4:**
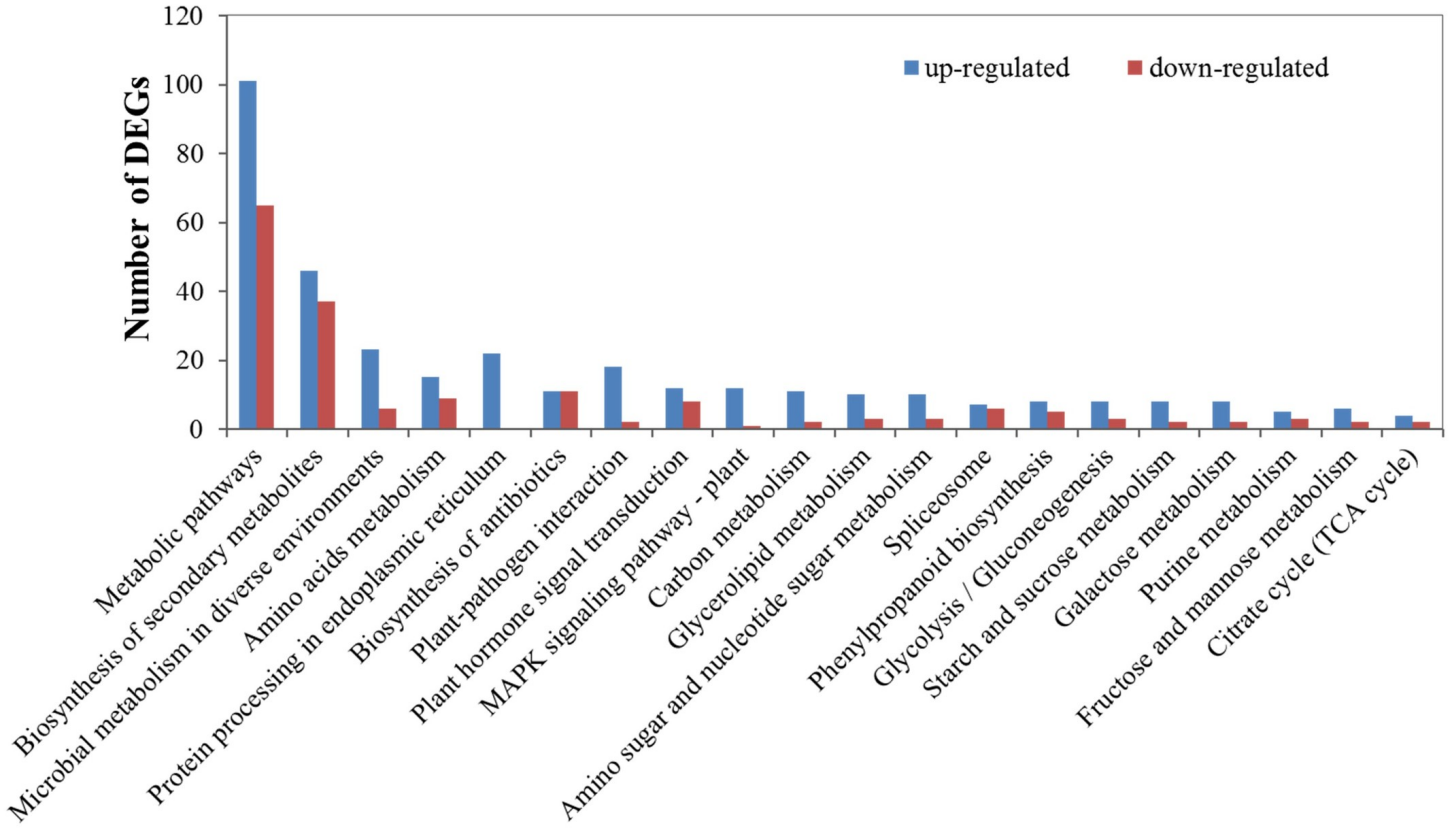
KEGG pathways enrichment of the DEGs during cold stress. Blue and red colors indicate up- and downregulated genes, respectively.

Under cold stress, the enrichment analysis of the DEGs based on the KEGG annotation showed that these genes mainly belonged to “metabolic pathways” (101 up- and 65 downregulated) and “biosynthesis of secondary metabolites” (46 up- and 37 downregulated). In most pathways, the number of upregulated genes exceeded the downregulated genes. In particular, some pathways related to sugar metabolism and amino acid metabolism were enriched with DEGs, including amino acid metabolism, glycolysis/gluconeogenesis, starch and sucrose metabolism, galactose metabolism, fructose and mannose metabolism, and the citrate cycle (TCA cycle) (Fig 4). In addition, it is commonly accepted that the plant MAPK signaling pathway is highly conserved and plays a central role in plant abiotic stress responses [51]. Here, KEGG annotation detected that the MAPK signaling pathway was strongly affected by cold stress. A total of 12 DEGs were upregulated, whereas only 1 DEG was downregulated (S3 Fig), suggesting that the MAPK signaling pathway might be involved in regulating cold-stress response in *X*. *sorbifolia*. The upregulated genes were MEKK1 (c29943.graph_c1), WRKY33 (c36168.graph_c0), VIP1 (c35571.graph_c1), PP2C (c24440.graph_c0), MAPKKK17_18 (c26611.graph_c0), CaM4 (c25814.graph_c0), FLS2 (c30712.graph_c0), WRKY22 (c26048.graph_c0), WRKY29 (c22982.graph_c1), RTE1 (c33293.graph_c0), ERF1 (c20551.graph_c0), and RbohD (c29720.graph_c0).

### GO annotation and classification

**Fig 5:**
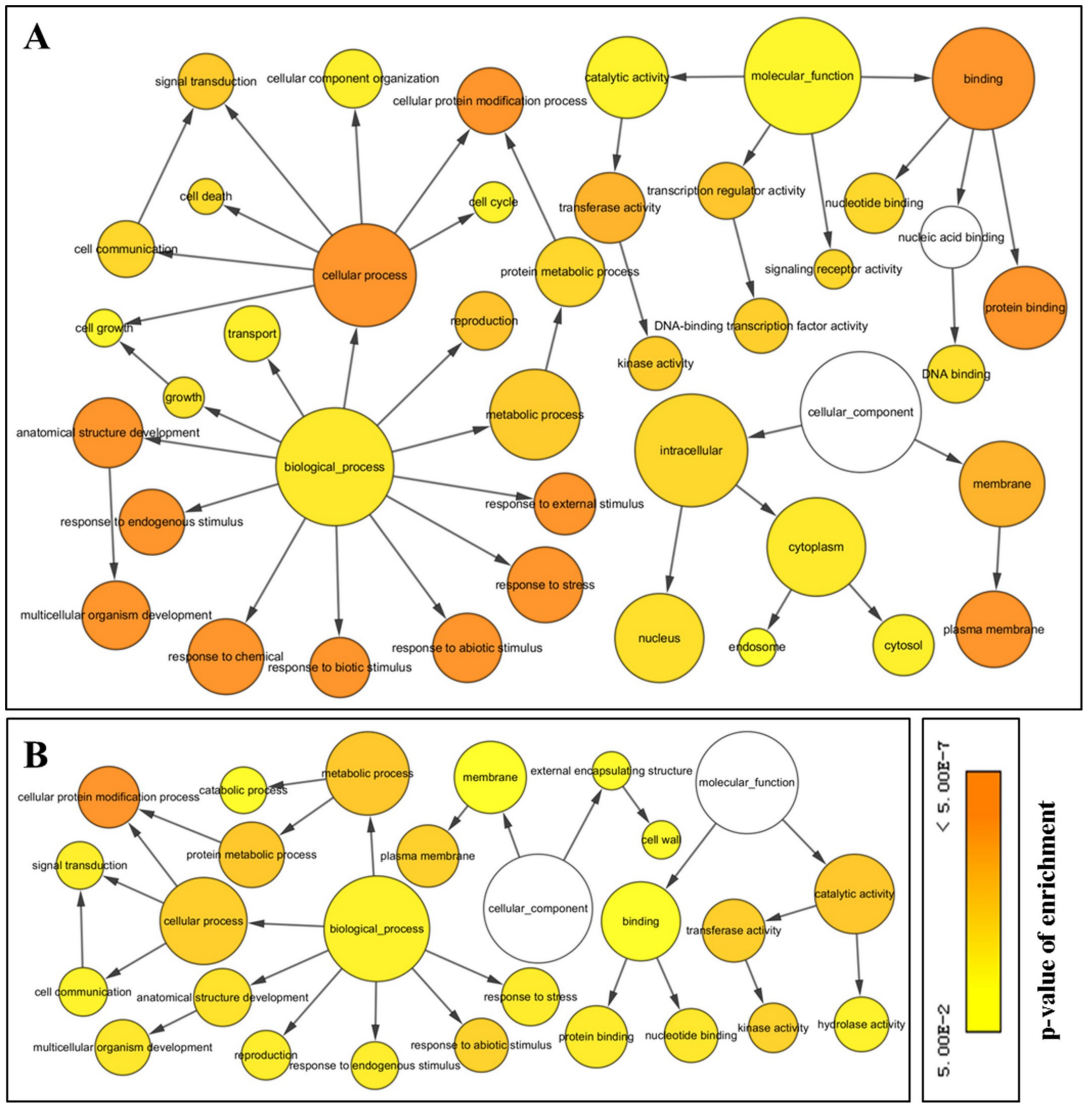
Networks of enriched GO terms in DEGs during cold stress. (A) Enriched GO terms from upregulated DEGs. (B) Enriched GO terms from downregulated DEGs. The lines represent the relationship between GO terms, with arrow from the higher ranking Go term pointing towards to the lower one. Circle size indicates the numbers of DEGs. Colors represent statistical significance.

All the significantly enriched GO terms among the DEGs were assembled into a GO map (Fig 5) to help visualize the interactive networks, and network features were presented in S4 Table. A total of 991 (out of 1,527) DEGs were assigned to the 3 main GO functional categories, with 563 up- and 428 downregulated genes. Across the network of GO terms enriched from upregulated DEGs, 196 DEGs (34.81%) were assigned into the largest category, “cellular component”, followed by “biological process” (186, 33.04%) and “molecular function” (181, 31.25%). A large number of DEGs were involved in some important GO terms that are known to be associated with cold tolerance in plants, such as “response to abiotic stimulus” (GO 9628, 8.88%), “response to endogenous stimulus” (GO 9719, 7.82%), and “response to stress” (GO 6950, 11.19%). For downregulated DEGs, the most frequent term for “biological process” was ‘cellular process’ (GO 9987, 21.03%); within “cellular component”, the most enriched term was related to ‘membrane’ (GO 16020, 13.08%); and the most enriched term within “molecular function” was ‘catalytic activity’ (GO 3824, 16.82%).

To further verify the reliability and accuracy of the RNA-Seq data, a total of 10 transcripts were selected for qRT-PCR analysis. The correlation analysis between qRT-PCR and RNA-Seq was measured by scatter plotting log2 (fold change), indicating that the expression patterns from qRT-PCR testing were highly consistent with the sequencing results (Pearson coefficient r^2^ = 0.88; S4 Fig).

### Metabolic profiling analysis

**Fig 6:**
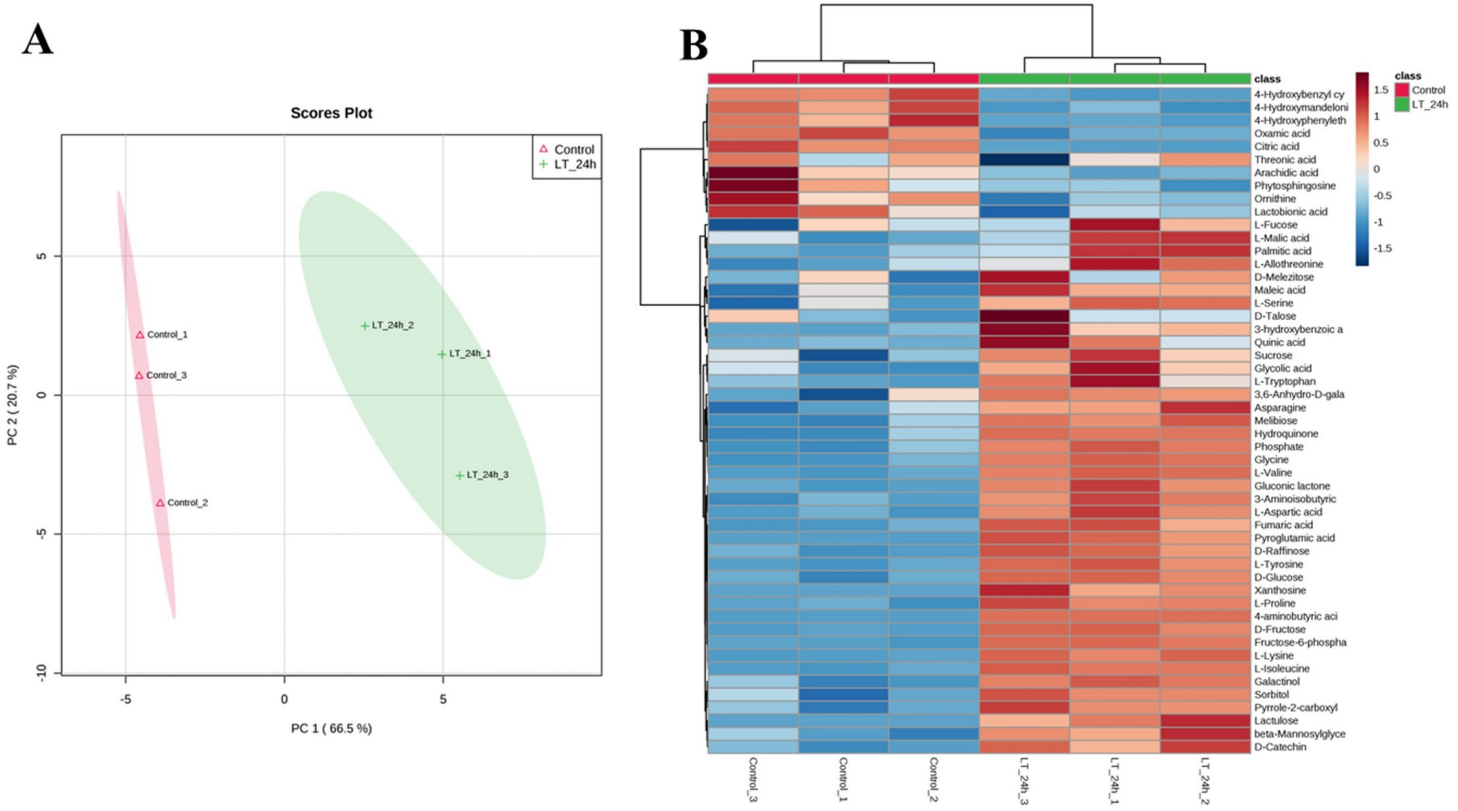
Metabolome analysis during cold stress in comparison with control. (A) PCA score plot based on metabolome data. PC1 and PC2 are plotted on the x- and y-axes, respectively. (B) HCA of the fold changes in all significantly changed metabolites between control and treatments.

Leaf extracts from the control and cold treatments were subjected to metabolic profiling using a gas chromatograph-mass spectrometer. In total, 262 metabolites were identified and quantified in all samples, including a number of primary and secondary metabolites, such as amino acids, sugars, organic acids, lipids, alkaloids, and terpenoids. Compared to the control, 51 metabolites were significantly altered under cold stress, with 41 upregulated and 10 downregulated. To investigate the accumulation patterns of these significantly changed metabolites in response to cold stress, principal components analysis (PCA) and hierarchical clustering analysis (HCA) were carried out. PCA suggested that the first two principal components (PC1 and PC2), which were extracted and represented a clear separation for 6 samples from the control and treatment groups, explained 87.2% of the total variation (Fig 6A). HCA also revealed the responses to cold stress at the metabolite levels, and successfully distributed the tested samples into two major clusters (Fig 6B). Particularly, some measured metabolites were exclusively significantly increased by cold stress, such as a 6.7-fold rise in fructose, 4.1-fold in glucose, 7.1-fold in lactulose, 4.1-fold in proline, 17.8-fold in pyroglutamic acid, 2.3-fold in quinic acid, 2.3-fold in 3-hydroxybenzoic acid, 4.2-fold in sorbitol, 409.2-fold in xanthosine, and 10.8-fold in hydroquinone (S5 Table). Through metabolic pathway mapping, these significantly changed metabolites were mainly annotated with amino acid metabolism, carbohydrate metabolism, aminoacyl-tRNA biosynthesis, and the TCA cycle, implying that these metabolic pathways play important roles in stress signaling and responses in *X*. *sorbifolia* (S5 Fig).

### Conjoint analysis of gene expression and metabolite levels involved in key biological pathways

**Fig 7:**
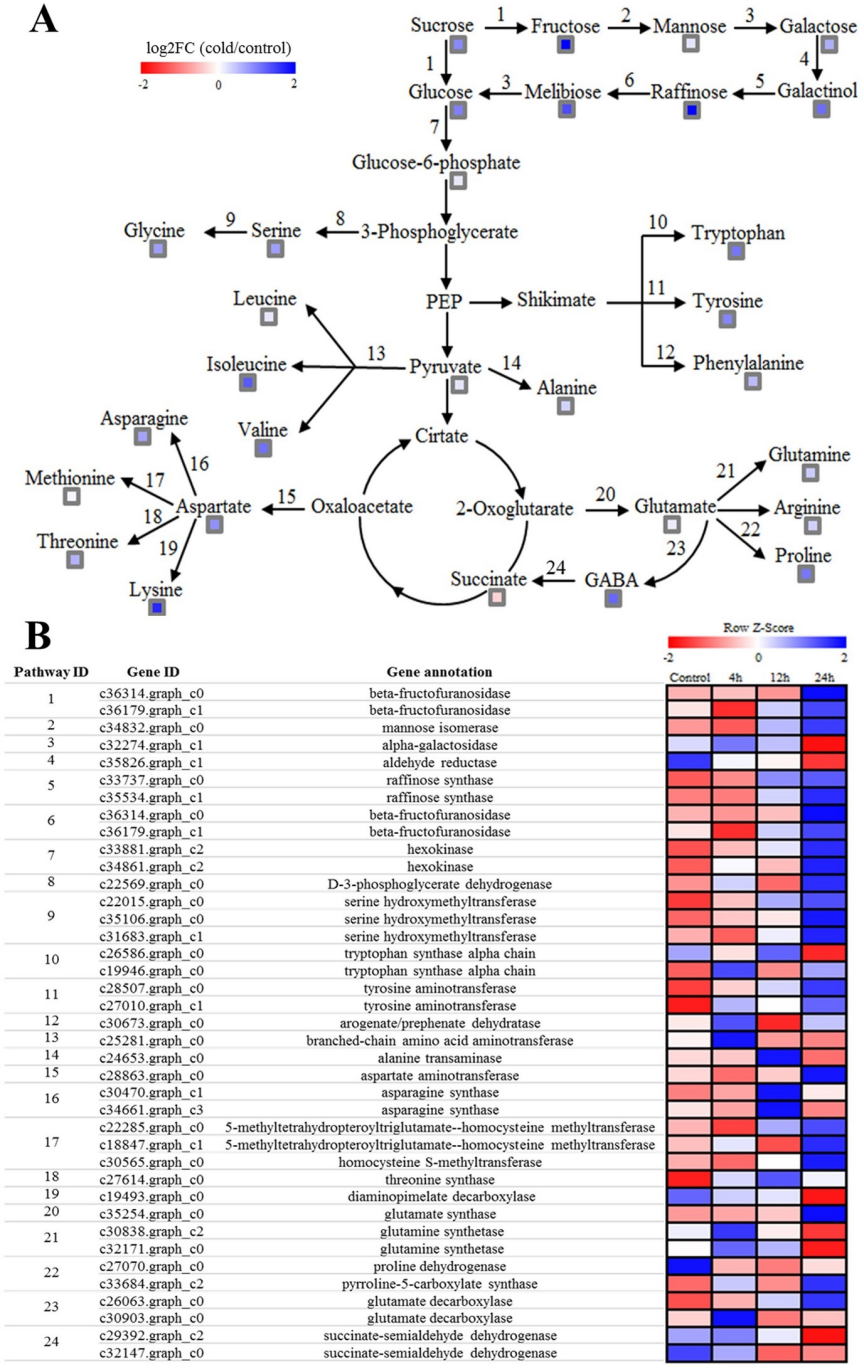
Overview of sugar and amino acid metabolic pathways during cold stress. (A) Changes of the metabolites associated with the pathways at 24 h after cold stress. (B) Expression patterns of the genes involved in metabolic pathways at different time points.

In plants, sugars and amino acids as osmotic substances are crucial regulatory elements during stress response. Interestingly, KEGG pathways involved in amino acid metabolism and sugar metabolism were enriched in both metabolite levels and gene expression under cold stress. Schematic diagrams of amino acid and sugar biosynthetic pathways of *X*. *sorbifolia* are presented in Fig 7A. The levels of most amino acids and sugars were significantly increased, suggesting that the accumulation of amino acids and sugars are important for the cold-stress response. Fig 7B shows the expression patterns of genes involved in the pathways at different time points. Within the pathways, cold stress resulted in the strong accumulation of fructose, glucose, raffinose, melibiose, galactinol, valine, glycine, lysine, isoleucine, tryptophan, serine, aspartate, proline, asparagine, and tyrosine. Most of the genes involved in the biosynthesis of these metabolites were upregulated, while there was a trend of downregulation among some genes of these pathways. The genes encoding beta-fructofuranosidase, mannose isomerase, raffinose synthase, and hexokinase (involved in sugar metabolism) were upregulated by cold stress; however, two genes encoding alpha-galactosidase and aldehyde reductase were downregulated. The gene encoding pyrroline-5-carboxylate synthase (P5CS, catabolizes the synthesis of proline) was upregulated; in contrast, one gene encoding proline dehydrogenase (catabolizes the degradation of proline) was downregulated. This was consistent with the elevated proline levels under cold stress. The lysine levels rose despite the downregulation of a related gene. The levels of other amino acids (such as serine, glycine, isoleucine, tryptophan) were increased within the amino acid metabolic pathways, and the expression levels of related genes involved in these pathways were upregulated at almost all time points. Two genes encoding succinate-semialdehyde dehydrogenase were downregulated, which was associated with reduced succinate levels under cold stress.

## Discussion

### Signaling transduction and regulation during cold stress

LT is initially recognized by the plasma membrane either based on alterations in membrane fluidity or with the aid of sensory proteins, such as Ca^2+^-permeable channels [52]. The membrane permeability of Ca^2+^ receptors was enhanced to allow more Ca^2+^ entry into the plasma after plants received cold signals, resulting in rapid accumulation of Ca^2+^ in the cytosol. Ca^2+^ is an important second messenger that plays a key role in the response to cold stress [53]. For instance, cold stress can cause an immediate rise in cytosolic free-Ca^2+^ concentration and activate Ca^2+^-permeable channels in *Arabidopsis* mesophyll cells [54]. Ca^2+^ sensors perceive intracellular changes in Ca^2+^ levels via phosphorylation and then transduce the signals in order to switch on downstream signaling processes for expression of cold-specific genes, which help in the adaptation of plants to cold stress [55]. The main intracellular Ca^2+^ sensors in plants are calmodulin (CaM), calcium-dependent protein kinases (CDPKs), calcineurin B-like proteins (CBLs), and their interacting protein kinases (CIPKs). In the present study, 12 DEGs were involved in the Ca^2+^ signaling pathway, of which 8 genes were upregulated and 4 genes were downregulated (S6A Fig). These upregulated genes contained 2 CaMs (c25814.graph_c0 and c29478.graph_c0), 2 CDPKs (c28536.graph_c1 and c32077.graph_c0), 2 CBLs (c35219.graph_c0 and c34566.graph_c3), and 2 CIPKs (c23992.graph_c0 and c28195.graph_c0), suggesting that they are associated with Ca^2+^ signaling and promote an increased intracellular Ca^2+^ concentration to activate various transcriptional cascades.

Besides calcium influx, abiotic stress can also cause the accumulation of ROS in the cytosol [18]. ROS has a signaling function that mediates defense gene activation by redox-sensitive transcription factors or via interaction with other signaling components, such as the MAPK cascade [56]. The MAPK cascade is a common signal transduction module that connects signals from receptors/sensors to nuclear and cellular responses [57, 58]. A total of 13 DEGs were involved in the plant-MAPK signaling pathway according to KEGG pathway annotation, and almost all DEGs were upregulated by cold stress (S6B Fig). It has been reported for *Arabidopsis* that the MAPKKK (MAP kinase kinase)-MAPKK (MAP kinase kinase)-MAPK cascade could be activated by ROS to enhance the cold resistance of plants [59]. Cold stress may also activate the MAPK cascade in *X*. *sorbifolia* to regulate gene expression, and MEKK1 and MAPKKK17_18 as cascade members were upregulated by cold stress. The MAPK cascade acts downstream of FLS2 to activate gene expression in *Arabidopsis* [60], and FLS2 was also upregulated by cold stress in *X*. *sorbifolia*. PP2C is a regulator of various signal transduction pathways that participate in stress response [61]. Evidences have indicated that PP2C controls the activation level of MAPK signaling [62], and it was upregulated by cold stress in our findings. The upregulation of ERF and CaM4 in the MAPK signaling pathway has been proposed as a secondary signal receptor of ethylene and Ca^2+^, respectively.

### Changes in ROS-scavenging system during cold stress

Intracellular ROS are thought to play a decisive role in regulating signal transduction events, but stress can cause ROS accumulation [63]. Excessive ROS enhance membrane lipid peroxidation (LPO) and disrupts membrane fluidity, resulting in electrolyte leakage (EL) [64]. Malondialdehyde (MDA) is also a peroxide lipid derivative, and its production can exacerbate membrane damage [65]. Here, Low-temperature treatment increased MDA concent and EL value in *X*. *sorbifolia*, indicating that cold stress caused probably the disruption of the plasma membrane. However, ROS homeostasis depends largely on ROS-scavenging systems under abiotic stress conditions [49, 66]. In the present study, we found a rise in the activity of ROS-scavenging enzymes (including SOD, CAT, and POD), suggesting a positive response when removing ROS generated by cold stress in *X*. *sorbifolia*. SOD is a scavenger of peroxide anions, which disproportionates the anions into H_2_O_2_ and O_2_ [63]. Meanwhile, POD and CAT can catalyze the conversion of H_2_O_2_ into oxygen and water [67]. The increase in SOD activity was lower than that of POD and CAT, potentially owing to POD and CAT having higher capacity for the decomposition of H_2_O_2_ generated by SOD. In addition, certain cold-responsive genes encoding anti-oxidative enzymes involved in the ROS-scavenging system, such as peroxidase (c32290.graph_c1 and else) and glutathione S-transferase (c35048.graph_c4), were strongly upregulated under cold stress, implying their significance in the ROS-scavenging system. Overall, the cold-induced enhancement of enzyme activity and gene expression might improve the ROS detoxification ability of *X*. *sorbifolia* against the detrimental effects of oxidative stress.

### Osmoprotectants metabolism during cold stress

Cold stress can induce osmotic stress in plant cells. Many plants maintain a stable osmotic pressure via the biosynthesis of osmoprotectants in the cold-stress response. Osmoprotectants are involved in the regulation of cellular water relations and reduce cellular dehydration [4]; moreover, they are non-toxic and accumulate at significant levels without upsetting plant metabolism [68]. In our study, strong cold-induced accumulation was observed in most of the amino acids, sugars, and sugar alcohols (S5 Table), while many of them can function as osmolytes in the cytoplasm under cold stress in plants [4]. Previous reports showed that various stress-responsive metabolites of plants were synthesized from amino acid metabolic pathways, suggesting that amino acid metabolism plays an important role in response to stress in plants [69]. For instance, proline accumulation in many plants is a common physiological response to abiotic stresses, such as LT, drought, and high salinity [70–72]. Proline is known to stabilize proteins, membranes, and subcellular structures, and protect cellular functions by scavenging ROS. The proline content was increased by cold stress and the important proline synthesis gene (P5CS, c33684.graph_c2) was upregulated, while the proline degradation gene (proline dehydrogenase, c27070.graph_c0) was downregulated (Fig 7B). Therefore, proline accumulation in *X*. *sorbifolia* under cold stress was based on co-regulation of P5C5 and proline dehydrogenase. Meanwhile, we detected the upregulation of several genes involved in amino acid metabolism and sugar metabolism, which was also consistent with the accumulation of special cold-responsive metabolites. In short, the changes observed at the transcriptomic and metabolic levels strongly revealed that accumulation of these cold-responsive metabolites by synthesis or metabolism pathways might contribute to cold-stress defense and thereby minimize cold damage.

### Transcription factors involved in response to cold stress

Many studies have shown that TFs are involved in transcriptional networks responding to abiotic stress in plants [73–75]. Consistent with findings in other plants, our transcriptomic data showed that most of the differentially expressed TFs belonged to the ERF, WRKY, NAC, bHLH, and MYB TF families, indicating the multiplicity and complexity of the regulatory pathways in response to cold stress [8, 76]. The ERF TF family was the largest class responding to cold stresses, with 13 upregulated and 2 downregulated DEGs. In particular, CBF/DREB from the ERF superfamily has been reported to play a vital role in regulating cold-responsive gene changes in many plants [77–79]. This was strengthened by the observation that a CBF TF (c28392.graph_c0) was significantly upregulated (132-fold), which played a direct role in activating the expression of downstream genes like COR. Currently, growing evidence has demonstrated that WRKY TFs are widely implicated in plant-stress responses [80, 81]. Notably, 13 WRKY family members in *X*. *sorbifolia* were all upregulated. Therefore, the overrepresentation of this TF family may indicate their importance in improving the resistance to cold response in *X*. *sorbifolia*. The plant-specific NAC TF family is involved in the regulation of multiple pathways, including plant development, hormone signaling, and stress response. RD26, which encodes a NAC TF, could remarkably enhance resistance to stress responses in *Arabidopsis* [82]. We found 8 genes encoding NAC TFs, and almost all were upregulated. These results emphasize that TFs play vital parts in enhancing cold resistance of *X*. *sorbifolia*. In addition, some of the MYB and bHLH TF family members were downregulated. MYB and bHLH TFs have also been reported to be involved in stress responses [83, 84], and they often interact with each other to control transcription [85]. Therefore, we deduced that these partner factors may be required in response to different cold conditions.

## Conclusions

In summary, cold stress caused significant changes in physiological, transcriptomic, and metabolomic levels in the leaves of *X*. *sorbifolia*. Based on the transcriptomic and metabolomics datasets, we successfully identified a number of candidate genes and metabolites that participate in crucial biological pathways during the cold-stress response. This was the first study to investigate the combined metabolomic and transcriptomic features of *X*. *sorbifolia* under cold stress, which reveals a complex regulation network and series of signaling mechanisms. These results provide useful insights into the tolerance mechanism of *X*. *sorbifolia* responding to cold stress.

## Supporting information

S1 Fig Pearson correlation analysis of sample replicates. (TIF)

S2 Fig Summary of active genes at different cold stress time points (A), and Venn diagrams showing overlap in numbers of expressed genes from control and treated samples (B). (TIF)

S3 Fig Plant-MAPK signaling pathway (Ko04016) map. Blue boxes indicated the up-regulated DEGs in leaf tissues of *X*. *sorbifolia* under cold stress.
(TIF)

S4 Fig Correlation analysis between qRT-PCR and RNA-Seq data based on log2 (fold change) of 10 selected genes. (TIF)

S5 Fig Metabolic pathways annotation of 51 significantly changed metabolites. (TIF)

S6 Fig Heatmap of DEGs involved in Ca^2+^ and MAPK signaling pathway in the three pairwise comparisons of control and stress treatments. (TIF)

S1 Table Primers used in qRT-PCR analysis. (XLS)

S2 Table Statistics of the unigene assembly results. (XLS)

S3 Table DEGs encoding transcription factor families. (XLS)

S4 Table Summary of gene ontology (GO) enrichment of DEGs in *X*. *sorbifolia* in response to cold stress. (XLS)

S5 Table The detail information of differentially changed metabolites in *X*. *sorbifolia* under cold stress. (XLS)
