# Supplementary figures and images for: Integrated transcriptomic and metabolomic analyses of yellow horn (Xanthoceras sorbifolia) in response to cold stress
Source: PLoS One. 2020 Jul 24;15(7):e0236588. doi: 10.1371/journal.pone.0236588 (PMC7380624; doi:10.1371/journal.pone.0236588)

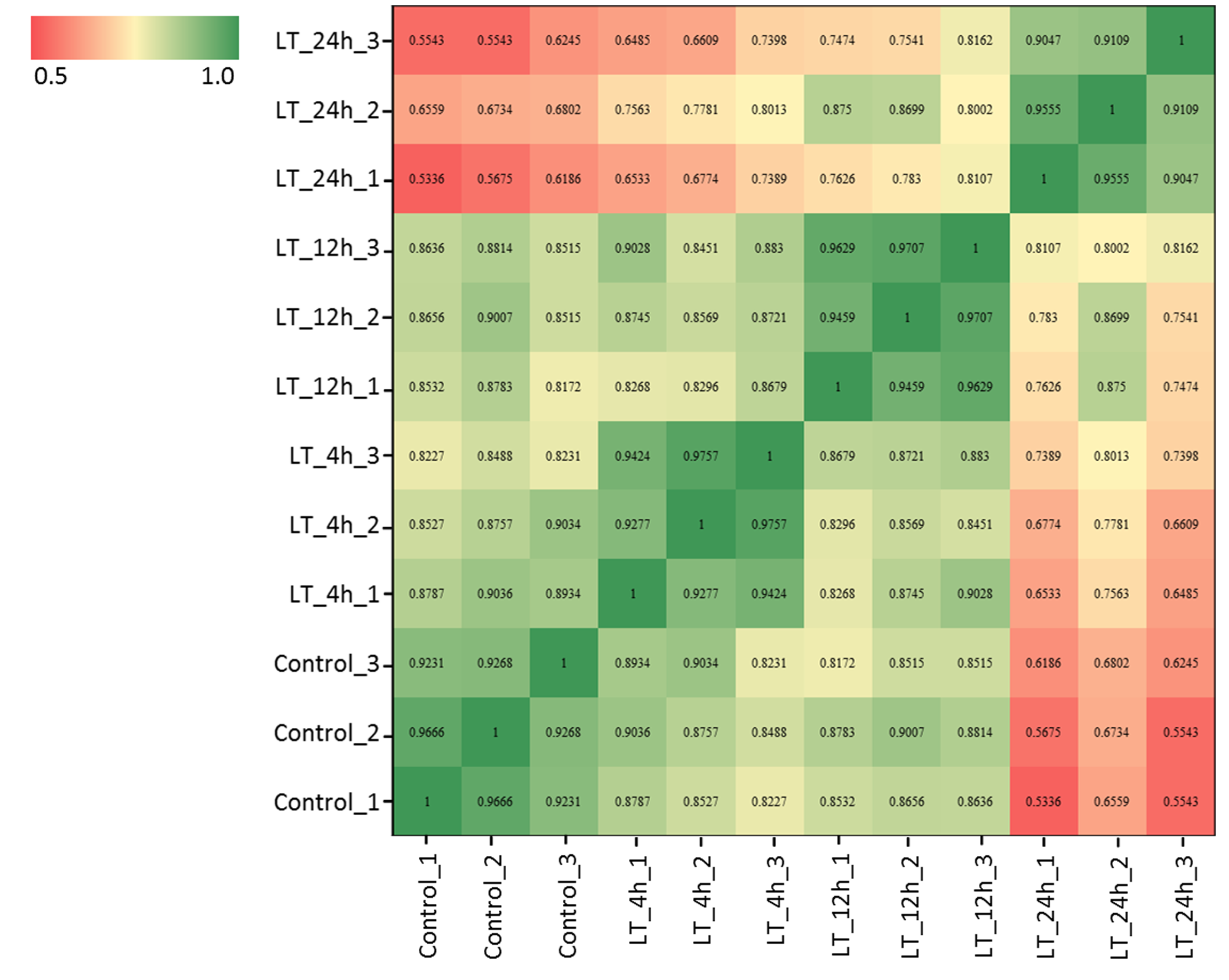

Supplement: S1 Fig — (TIF) [file pone.0236588.s001.tif]

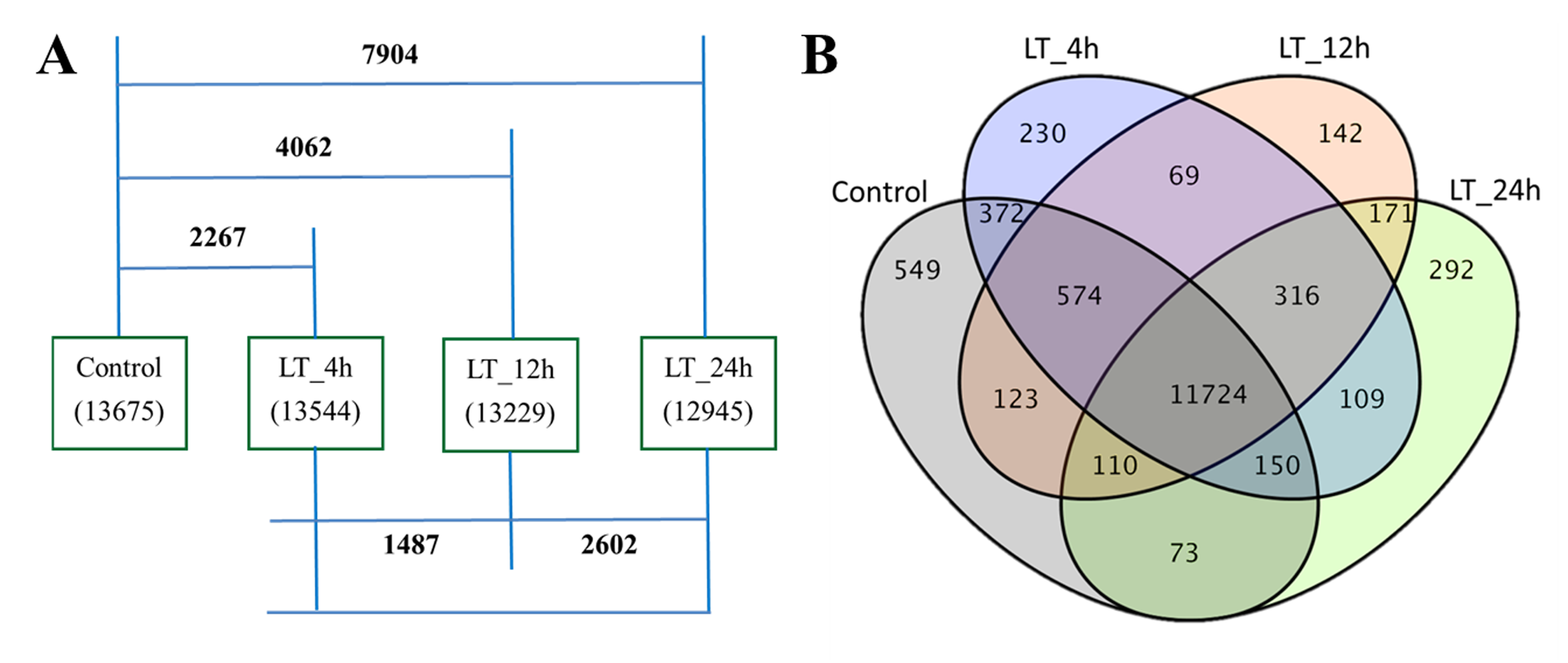

Supplement: S2 Fig — (TIF) [file pone.0236588.s002.tif]

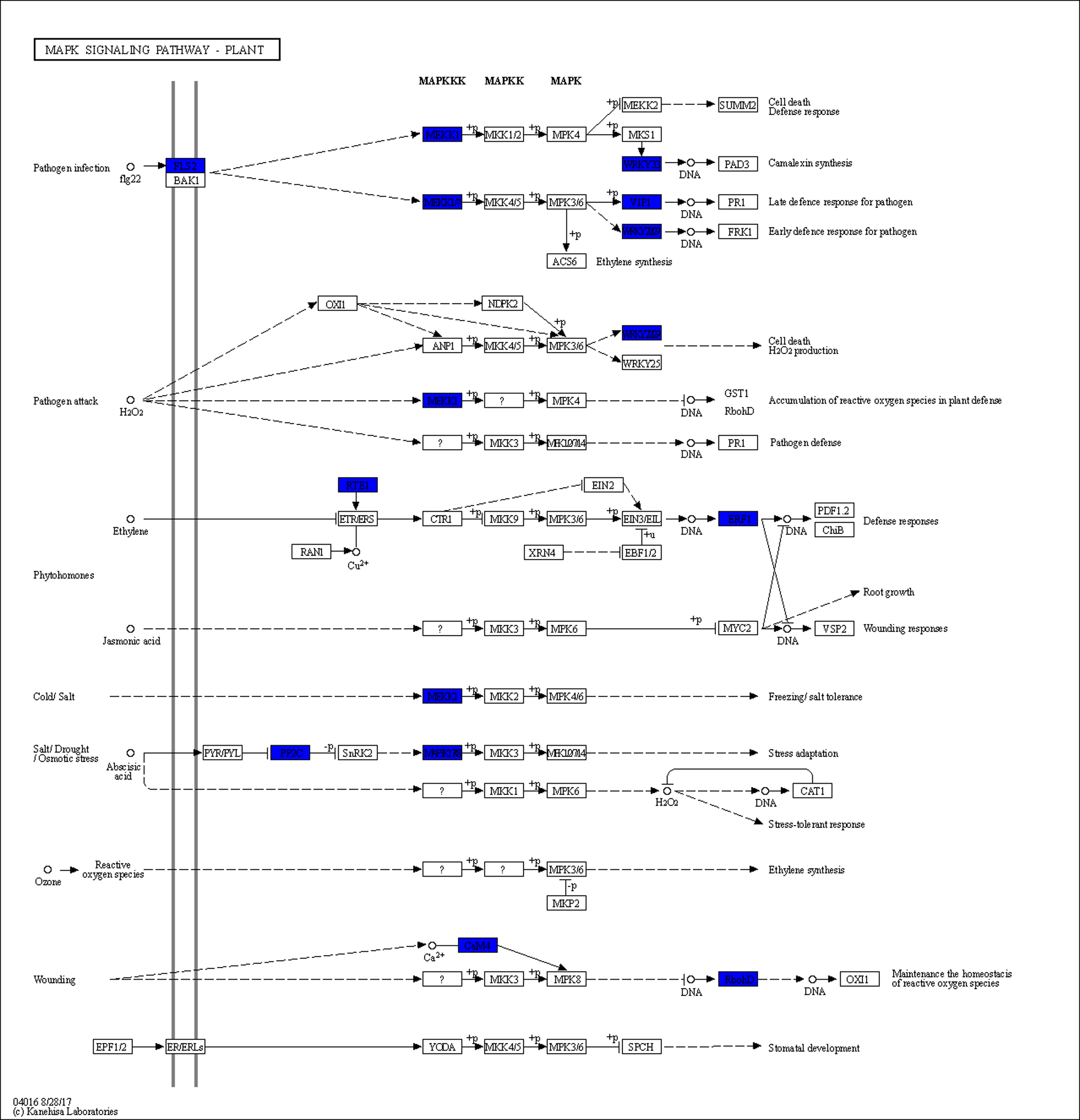

Supplement: S3 Fig — Blue boxes indicated the up-regulated DEGs in leaf tissues of X. sorbifolia under cold stress. (TIF) [file pone.0236588.s003.tif]

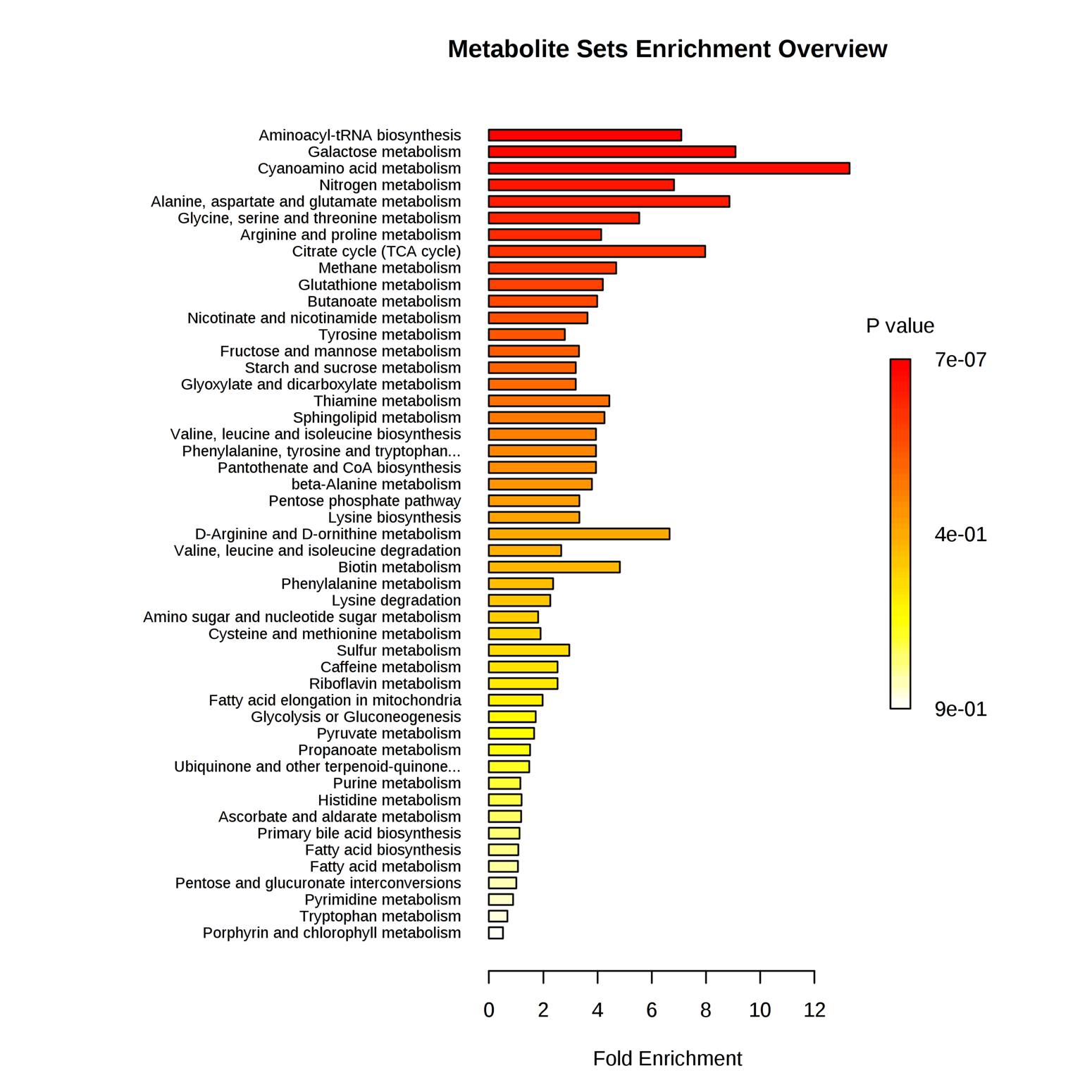

Supplement: S5 Fig — (TIF) [file pone.0236588.s005.tif]

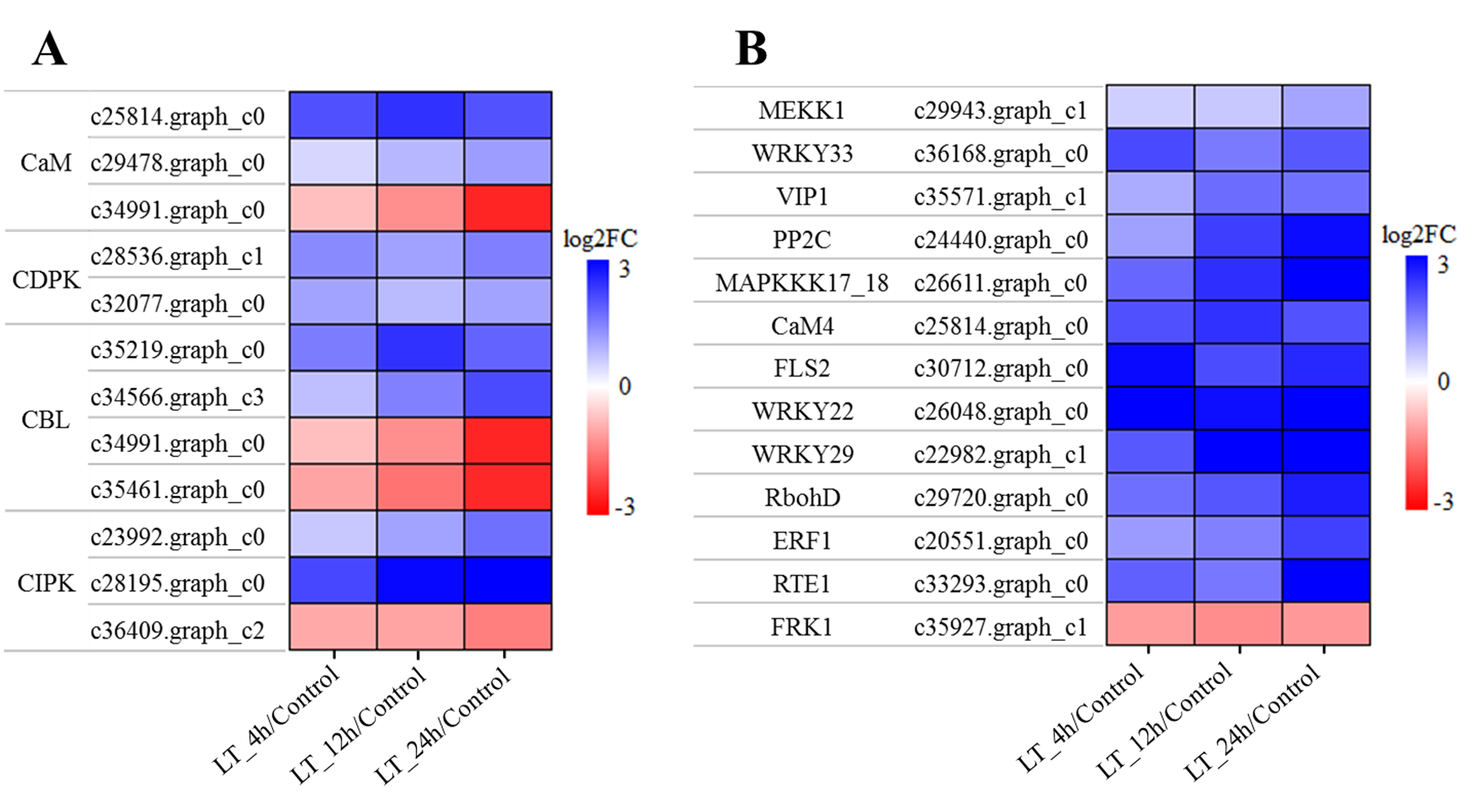

Supplement: S6 Fig — (TIF) [file pone.0236588.s006.tif]
